# The Increment of Choroidal Thickness in Euthyroid Graves' Ophthalmopathy: Is It an Early Sign of Venous Congestion?

**DOI:** 10.1155/2018/5891531

**Published:** 2018-08-28

**Authors:** Eylem Cagiltay, Fahrettin Akay, Ozgur Demir, Erdinç Aydın, Berkay Akmaz, Baris Pamuk

**Affiliations:** ^1^Department of Endocrinology and Metabolic Diseases, Sultan Abdulhamid Han Education and Research Hospital, Medical Sciences University, Istanbul, Turkey; ^2^Department of Opthalmology, Ataturk Education and Research Hospital, Katip Celebi University, Izmir, Turkey; ^3^Department of Endocrinology and Metabolic Diseases, Ankara University, Ankara, Turkey; ^4^Department of Endocrinology and Metabolic Diseases, Ataturk Education and Research Hospital, Katip Celebi University, Izmir, Turkey

## Abstract

**Objective:**

Clinical manifestations of Graves' ophthalmopathy (GO) are caused by the overcompression of orbital tissues within the restricted orbital bone cavity. Impaired ocular blood flow may disrupt the retinal microstructure and functions. In this study, we aimed to investigate the macular and choroidal thickness changes in GO compared with healthy subjects.

**Materials and Methods:**

The study group comprised 50 adult patients with previously diagnosed Graves' disease with ophthalmopathy who were on antithyroid treatment. For the assessment of GO activity, the VISA (vision, inflammation, strabismus, and appearance) inflammatory score was used. When euthyroidism was achieved without side effects, the patients were referred to the ophthalmology clinic for spectral-domain optical coherence tomography (SD-OCT) evaluation.

**Results:**

Subfoveal, mean, and temporal choroidal thicknesses were increased significantly in the study group according to the controls. The mean choroidal thickness was elevated.

**Conclusions:**

This elevation is because of the intraorbital inflammation even in this nonsevere GO group. Choroidal thickness might be affected from the venous obstruction and congestion in patients with GO. The elevation of the choroidal thickness might be an early sign of venous congestion that occurs before the elevation of intraocular pressure.

## 1. Introduction

Graves' disease (GD) is an autoimmune disorder which causes different impairments in the thyroid gland, orbit, and pretibial skin. The incidence of GD is approximately 5% in the population [1]. Graves' ophthalmopathy (GO) may develop in nearly 25–50% of patients with GD. Clinical signs of GO include eyelid retraction, exophthalmos, strabismus, and optic neuropathy. Stimulatory antibodies against the thyroid-stimulating hormone receptor (TSH-R) activate the thyroid gland which leads to hyperthyroidism. Cytokines, chemokines, antibodies, T cells, and B cells are thought to be responsible from some pathogenic mechanisms. Also, they give rise to inflammation at the orbital connective tissue and extraocular muscles [2]. Clinical course of GO has active and inactive inflammatory cycles. TSH-R autoantibodies correlate positively with the clinical severity of GO [3]. Orbital inflammation, which activates the fibroblasts, causes the expansion of the orbital adipose tissue and extraocular muscles by the synthesis of hyaluronan and glycosaminoglycans [4].The globe is pushed forwardly due to this overproduction and adipose hyperplasia which called as proptosis [3]. Clinical manifestations of GO are caused by the overcompression of orbital tissues within the restricted orbital bone cavity.

Ocular blood flow is very important for a healthy retina and other ocular tissues. Several authors reported ocular hemodynamic alterations in GO with dissimilar techniques [5–7]. Hyperthyroidism, systemic blood pressure, orbital inflammation, and intraocular pressure may affect the ocular hemodynamics [8]. Some researchers reported the decrease in the superior ophthalmic vein blood flow velocity, and this may increase the severity of GO and optic neuropathy [9, 10]. From the ophthalmic artery, 70% of the total ocular blood flow is directed to the choroidal tissue. Impaired choroidal blood flow may disrupt the retinal microstructure and functions. Spectral-domain optical coherence tomography (SD-OCT) provides the measurement of the microstructure of the retina and choroidal vessels noninvasively. Early recognition of retinal and choroidal changes may alert the physicians for preventing ocular complications of GO. In this study, we aimed to investigate the choroidal thickness changes in euthyroid GO patients compared with healthy subjects.

## 2. Materials and Methods

This present study was designed as a prospective observational clinical cross-sectional case-control study and performed in İzmir Katip Çelebi University Atatürk Training and Research Hospital. It was approved by the institutional ethics committee and conducted with the tenets of the Declaration of Helsinki. Patients with GO were referred to the ophthalmology department routinely by the endocrinology clinic for ocular examinations. The study group comprised 50 adult patients with previously diagnosed Graves' disease with ophthalmopathy who were on regular antithyroid treatment. The control group comprised 50 healthy age- and sex-matched subjects selected among healthy volunteers. When euthyroidism (defined as TSH, 0.35–4.94 *μ*IU/mL; T4, 0.7–1.48 ng/dL) was achieved without side effects, the patients were referred to the ophthalmology department for clinical and OCT evaluations. GO was confirmed by an experienced ophthalmologist (FA) according to the clinical findings (lid-lag, eye movements, proptosis, scleral show, and strabismus), characteristic magnetic resonance images, and other clinical signs. We used the VISA (vision, inflammation, strabismus, and appearance) inflammatory score for the assessment of GO activity, (Table 1) and VISA ≥5/8 showed active GO [11].

Patients and controls with ocular or other systemic diseases and smokers were excluded. The blood pressure measurements were performed after subjects had been seated for at least 20 minutes. Each subject underwent full ophthalmic examination including refraction, best spectacle-corrected visual acuity (BCVA), intraocular pressure (Goldmann applanation tonometry), axial length (AL) by biometry (OcuScan; Alcon, Fort Worth, TX, USA), and 90-D lens funduscopy. The exclusion criteria included cataract, diabetic retinopathy, macular edema, any type of previous retinal treatment (macular laser photocoagulation, vitrectomy, intravitreal steroids, and/or antiangiogenic agents), history of any ocular surgery, spherical equivalent >3 D, glaucoma, ocular hypertension, uveitis, other retinal diseases, and neurodegenerative disease (e.g., Alzheimer, Parkinson, and dementia).

**Figure 1:**
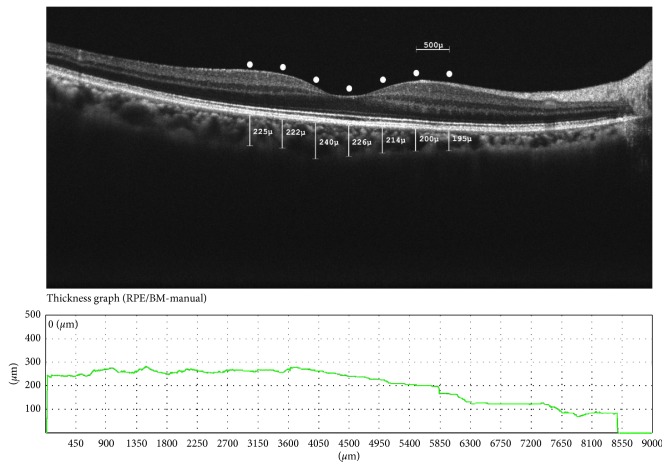
A represantative choroidal thickness in Graves' ophthalmopathy.

All participants were examined with SD-OCT (Retina scan, RS-3000; Nidek, Japan) after pupillary dilation with tropicamide 1% (Tropamid 1%; Bilim İlaç, Turkey). At least three images were taken, and the one with the highest signal strength (≥8) was chosen for analysis. A macula line raster scan was used to measure the choroidal thickness. Distance between the retinal pigment epithelial-Bruch's membrane layer (automatically drawn) and the sclerochoroidal interface drawn by an examiner (FA) who was blinded to the diagnosis of the participants was determined. The choroidal thickness was measured at 7 different points: at the subfovea; at 500 *μ*m, 1000 *μ*m, and 1500 *μ*m temporal to the fovea; and at 500 *μ*m, 1000 *μ*m, and 1500 *μ*m nasal to the fovea (Figure 1). Only one eye of each participant demonstrated with the most inflammatory activity, and the fellow eye was assessed for statistical analysis. All SD-OCT measurements were performed between 9:00 and 11:00 AM.

All statistical data were analyzed using SPSS version 21.0 (IBM Cor., USA). Values were expressed as mean ± standard deviation. The normality of the values was analyzed by using Kolmogorov–Simirnov test. The independent samples *t*-test or Mann–Whitney *U* test was used according to the normality scores. The Pearson correlation coefficient was used for correlation between factors. The multivariate regression test was used to analyze factors with CT in patients with GO. Finally, the results were considered significant at *p* < 0.05.

## 3. Results

**Figure 2:**
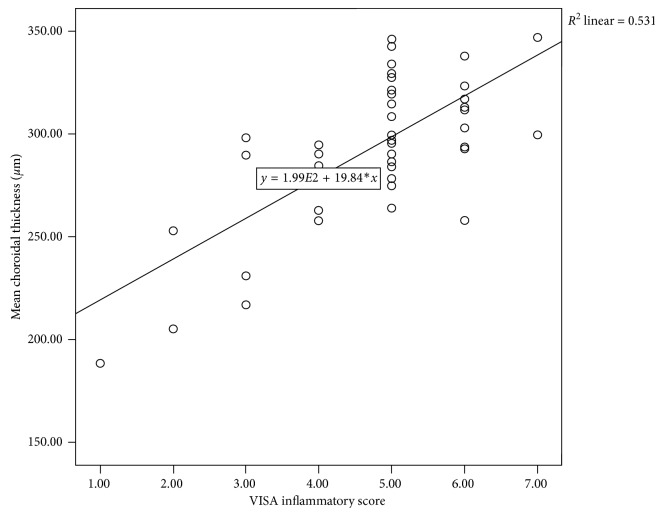
The correlation plot of the mean choroidal thickness and the VISA inflammatory score in patients with Graves' ophthalmopathy positively correlated with the VISA score (*p*=0.001; *R*^2^=0.531).

The mean age of the study group and controls were 36.20 ± 5.3 years and 35.42 ± 6.0 years, respectively (*p*=0.50). Thirty-nine patients were male and 11 patients were female in the GO group. There was no statistical difference in gender between the groups (*p*=0.30). Other characteristics of the groups are demonstrated in Table 2. As expected, exophthalmometry measurements in the GO group were increased according to the controls, and also, there was a significant difference between the groups (*p*=0.002). There were no significant differences between the groups with other parameters. IOP was within normal limits in both groups. BCVA was not affected from ophthalmopathy in the study group. Subfoveal, mean, and temporal choroidal thicknesses were increased in the study group according to the controls. And also, there were statistically significant differences. But nasal peripapillary choroidal thicknesses increased minimally in the study group, and there was no statistical difference according to the healthy subjects (Table 3). The mean choroidal thickness was increased correlated with the VISA score in Pearson analysis (*p*=0.001, *r*=0.646) (Figure 2).The mean choroidal thickness was accepted as a dependent variable, and exophthalmometry, disease duration, mean blood pressure, and axial length parameters were accepted as independent variables in multivariate analysis. There were no significant correlations between the factors (*p* > 0.05).

## 4. Discussion

Choroidal blood circulation might be associated with the pathogenesis of retinal, retina pigment epithelium, and optic nerve diseases. SD-OCT is a novel and noninvasive method for objective evaluation of the choroidal vascular structure and retina. Correlation between CT and the in vivo choroidal function remains unclear, but it is the best available technique [12]. Some of Graves' patients are affected in both extraocular muscles and the orbital adipose tissue in pathogenesis of the GO. The expanded orbital tissues in GO push the globe forwardly and disorder the intraorbital blood circulation. It was found that superior ophthalmic vein (SOV) blood flow was lower in active GO [7]. And also, it is supported that decreased SOV circulation may worsen the orbital inflammation in active GO [13]. In our study, subfoveal, temporal regions, and mean choroidal thicknesses were elevated in the study group according to the healthy subjects. These findings show the choroidal thickness might be elevated from the venous circulation congestion in GO. The elevation of the choroidal thickness might be an early sign of venous congestion that occurs before the elevation of IOP.

Choroidal thickness values are in normal subjects approximately 250 ± 400 *μ*m at the subfoveal point and decrease in the temporal and nasal regions [14, 15]. Choroidal circulation may be affected by different factors like age, systemic arterial blood pressure, diurnal variation, cigarette smoking, and heart failure [16–18]. In the present study, the systemic arterial blood pressure measurement was normal in both groups. There was no difference between groups according to the age factor. Other systemic diseases and smoking were excluded from the study which affects the CT.

Inflammation of the eye affects the choroidal vascular structure. Some authors reported many ocular inflammatory diseases that affect the choroid like Vogt–Koyonagi–Harada (VKH), sarcoidosis, Behçet's disease, inflammatory bowel disease, and posterior scleritis before [19–22]. According to these different studies in the acute phase, choroidal thickness usually increases, but recurrent inflammatory changes decrease the CT and causes the atrophic changes which results as choroidal thinning. As known, both cellular and humoral immunity play a role in the autoimmune reactions of GO. Thyrotropin receptors are expressed in orbital fibroblasts and initiate the disease pathologic mechanisms. Macrophages, CD4+ T cells, CD8+ T cells, and B cells infiltrate the perivascular regions in orbital tissues [23]. Inflammatory reactions in the orbital fat tissue and rectus muscles can indirectly affect the choroidal vascular network by disturbing the orbital blood circulation. Inflammation of orbital soft tissues may lead to proptosis. The mean value of exopthalmometry was a little over in the study group according to the controls and statistically significant. Proptosis may increase IOP. The IOP was measured to be minimally higher in the study group than the controls, but there was no significant difference in both groups.

## 5. Conclusion

In our study, as seen in Figure 2, increase in VISA scores and choroidal thickness is directly proportional. As the inflammation in the orbital tissues increases, the VISA score increases. This elevation in choroidal thickness can be explained by the increase in vascular permeability and vascular dilatation caused by inflammatory factors. Çalışkan et al. reported that inflammation affects choroidal thickness in GO patients [24]. Özkan et al. have done a study showing that the subject has increased choroidal thickness correlated with clinical activity scores (CAS) [25]. These two studies support our similar findings. Also, hyperthyroidism has been reported for the increased risk for deterioration of GO [26]. To avoid systemic effects of hyperthyroidism, ophthalmic evaluations were made in the euthyroid period, and this is an advantage for our study.

Our study has a number of advantages over similar ones done earlier. Firstly, all patients were in the euthyroid period and none of them have received radioactive iodine treatment, and they were a little younger in age. There are some limitations in our study. This clinical study was designed as cross-sectional. If designed as a prospective study, more information about the pathophysiologic course of the disease could be obtained. More data can be obtained about changes in the choroidal thickness by performing SD-OCT measurements in the active and inactive phases of ophthalmopathy. And also, the euthyroid and hyperthyroid stages can be compared. Another major deficiency of our study is the inability to observe choroidal changes over time in older and inactive ophthalmopathy patients.

As a result, an overall increase in the choroidal thickness was detected in active GO patients in the present study. Consequently, this is thought to be caused by compression of the orbital venous circulation and inflammation of the soft tissues. With the development of better OCT devices in the future, the choroidal vascular network will be better visualized. However, it is to be understood that the thyroid ophthalmopathy is further damaging to which layer of choroid. This will allow to better understand the damage mechanisms to ocular tissues and to develop successful treatment protocols.

## Figures and Tables

**Table 1:** VISA inflammatory score.

Clinical findings	Score
Orbital pain (none, at rest, with gaze)	0–2
Chemosis	0–2
Eyelid edema	0–2
Conjunctival injection	0-1
Eyelid injection	0-1
Total	0–8

**Table 2:** Characteristics of patients in the study and control groups.

Characteristics	Study group *n*=50	Control group *n*=50	*p* value
Age (years)	36.2 ± 5.3	35.4 ± 6.0	0.50^*∗*^
Males, *n* (%)	39/11	43/7	0.30^*∗*^
BCVA, logmar	−0.006 ± 0.02	−0.008 ± 0.02	0.69^*∗*^
IOP (mmHg)	16.1 ± 2.2	15.9 ± 1.9	0.86^*∗∗*^
Axial length (mm)	22.4 ± 0.7	22.7 ± 0.6	0.20^*∗∗*^
Spherical equivalent (dpt)	−0.35 ± 0.89	−0.33 ± 0.63	0.52^*∗*^
Exophthalmometry (mm)	18.7 ± 2.2	17.4 ± 0.9	**0**.**002**^*∗*^
BMI	24.2 ± 1.9	24.3 ± 1.6	0.69^*∗∗*^
MBP (mmHg)	88.9 ± 4.3	88.3 ± 4.7	0.53^*∗∗*^
Disease duration (years)	3.1 ± 1.7	N/A	—
VISA inflammatory score	4.7 ± 1.3	N/A	—

BCVA = best corrected visual acuity; logMar = logaritm of the minimum angle of resolution; IOP = intraocular pressure; BMI = body mass index; MBP = mean blood pressure; N/A = not applicable; ^*∗*^Mann–Whitney *U* test; and ^*∗∗*^Independent samples *t*-test.

**Table 3:** Choroidal thickness differences between the groups.

Choroidal thickness location	Study group *n*=50	Control group *n*=50	*p* value^*∗*^
Subfoveal	307.1 ± 30.2	275.5 ± 34.2	*p* < 0.001
Temporal, 500 *µ*m	314.3 ± 31.6	274.2 ± 34.9	*p* < 0.001
Temporal, 1000 *µ*m	312.2 ± 31.3	272.9 ± 33.2	*p* < 0.001
Temporal, 1500 *µ*m	303.9 ± 28.3	267.6 ± 31.8	*p* < 0.001
Nasal, 500 *µ*m	296.4 ± 28.9	270.8 ± 34.7	*p* < 0.001
Nasal, 1000 *µ*m	278.5 ± 30.4	263.5 ± 34.1	*p*=0.023
Nasal, 1500 *µ*m	261.8 ± 32.9	253.1 ± 34.7	*p*=0.20
Mean	296.3 ± 28.1	268.2 ± 33.1	*p* < 0.001

^*∗*^Independent samples *t*-test.

## Data Availability

The data used to support the findings of this study are the personal data of the patients that have been taken with written informed consent. Data are available from the corresponding author for researchers who meet the criteria for access to confidential data.

## References

[1] Antonelli A., Ferrari S. M., Corrado A., Di Domenicantonio A., Fallahi P. (2015). Autoimmune thyroid disorders. *Autoimmunity Reviews*.

[2] Prabhakar B. S., Bahn R. S., Smith T. J. (2003). Current perspective on the pathogenesis of Graves’ disease and ophthalmopathy. *Endocrine Reviews*.

[3] Bahn R. S. (2015). Current insights into the pathogenesis of Graves’ ophthalmopathy. *Hormone and Metabolic Research*.

[4] Kazim M., Goldberg R. A., Smith T. J. (2002). Insights into the pathogenesis of thyroid-associated orbitopathy: evolving rationale for therapy. *Archives of Ophthalmology*.

[5] Tsai C. C., Kau H. C., Kao S. C. (2005). Pulsatile ocular blood flow in patients with Graves’ ophthalmopathy. *Eye*.

[6] Kurioka Y., Inaba M., Kawagishi T. (2001). Increased retinal blood flow in patients with Graves’ disease: influence of thyroid function and ophthalmopathy. *European Journal of Endocrinology*.

[7] Alp M. N., Ozgen A., Can I., Cakar P., Gunalp I. (2000). Colour doppler imaging of the orbital vasculature in Graves’ disease with computed tomographic correlation. *British Journal of Ophthalmology*.

[8] Perri P., Campa C., Costagliola C., Incorvaia C., D’Angelo S., Sebastiani A. (2007). Increased retinal blood flow in patients with active Graves’ ophthalmopathy. *Current Eye Research*.

[9] Konuk O., Onaran Z., Ozhan Oktar S., Yucel C., Unal M. (2009). Intraocular pressure and superior ophthalmic vein blood flow velocity in Graves’ orbitopathy: relation with the clinical features. *Graefe’s Archive for Clinical and Experimental Ophthalmology*.

[10] Onaran Z., Konuk O., Oktar S. O., Yucel C., Unal M. (2014). Intraocular pressure lowering effect of orbital decompression is related to increased venous outflow in Graves orbitopathy. *Current Eye Research*.

[11] Dolman P. J., Rootman J. (2006). VISA classification for graves orbitopathy. *Ophthalmic Plastic and Reconstructive Surgery*.

[12] Novais E. A., Badaro E., Allemann N. (2015). Correlation between choroidal thickness and ciliary artery blood flow velocity in normal subjects. *Ophthalmic Surgery, Lasers and Imaging Retina*.

[13] Monteiro M. L., Moritz R. B., Angotti Neto H., Benabou J. E. (2011). Color Doppler imaging of the superior ophthalmic vein in patients with Graves’ orbitopathy before and after treatment of congestive disease. *Clinics*.

[14] Karapetyan A., Ouyang P., Tang L. S., Gemilyan M. (2016). Choroidal thickness in relation to ethnicity measured using enhanced depth imaging optical coherence tomography. *Retina*.

[15] Branchini L. A., Adhi M., Regatieri C. V. (2013). Analysis of choroidal morphologic features and vasculature in healthy eyes using spectral-domain optical coherence tomography. *Ophthalmology*.

[16] Akay F., Gundogan F. C., Yolcu U., Toyran S., Uzun S. (2016). Choroidal thickness in systemic arterial hypertension. *European Journal of Ophthalmology*.

[17] Spaide R. F. (2009). Age-related choroidal atrophy. *American Journal of Ophthalmology*.

[18] Tan K. A., Gupta P., Agarwal A. (2016). State of science: choroidal thickness and systemic health. *Survey of Ophthalmology*.

[19] Baltmr A., Lightman S., Tomkins-Netzer O. (2014). Examining the choroid in ocular inflammation: a focus on enhanced depth imaging. *Journal of Ophthalmology*.

[20] Ishikawa S., Taguchi M., Muraoka T., Sakurai Y., Kanda T., Takeuchi M. (2014). Changes in subfoveal choroidal thickness associated with uveitis activity in patients with Behcet’s disease. *British Journal of Ophthalmology*.

[21] Fong A. H., Li K. K., Wong D. (2011). Choroidal evaluation using enhanced depth imaging spectral-domain optical coherence tomography in Vogt-Koyanagi-Harada disease. *Retina*.

[22] Onal I. K., Yuksel E., Bayrakceken K. (2015). Measurement and clinical implications of choroidal thickness in patients with inflammatory bowel disease. *Arquivos Brasileiros de Oftalmologia*.

[23] Wang Y., Smith T. J. (2014). Current concepts in the molecular pathogenesis of thyroid-associated ophthalmopathy. *Investigative Ophthalmology and Visual Science*.

[24] Çalışkan S., Acar M., Gurdal C. (2017). Choroidal thickness in patients with Graves’ ophthalmopathy. *Current Eye Research*.

[25] Özkan B., Kocer C. A., Altintas O., Karabas L., Acar A. Z., Yuksel N. (2016). Choroidal changes observed with enhanced depth imaging optical coherence tomography in patients with mild Graves orbitopathy. *Eye*.

[26] Wiersinga W. M., Bartalena L. (2002). Epidemiology and prevention of Graves’ ophthalmopathy. *Thyroid*.

